# Transplantation of Skin for the Cure of Ectropium1Read before the Chicago Medical Society, February 27.

**Published:** 1889-03

**Authors:** H. Gradle

**Affiliations:** 63 State Street


### REPORT OF A CASE OF TRANSPLANTATION OF SKIN FOR THE CURE OF ECTROPIUM.[1]

BY H. GRADLE, M.D.

The young lady whom I wish to exhibit to you to-night, in order to show the result of an ectropium operation, has had ectropium of the lower lid of the eye since her childhood, due at that time to some form of ulceration, probably lupus. Ten years ago some form of operation was performed without beneficial result. When first seen the lower lid was everted throughout almost its entire extent, the conjunctiva exposed, and, of course, in an abnormal and irritated condition. The scar underneath the lower lid, extending externally to the external canthus, had pulled the skin of the lid down and had caused it to adhere to the inferior rim of the orbit.

On September 24, 1888, I made the usual form of ectropium operation by cutting through the skin in a line parallel to the ciliary border and some three millimeters below it, and liberating it from the scar by undermining until the lid could be replaced satisfactorily. Into the gaping wound I then transplanted a pediculated flap taken from the healthy skin on the malar prominence immediately adjoining the external canthus of the eye. The flap was some twelve to fifteen millimeters wide, but, of course, shrunk at once so as seem about right for the width of the wound, which varied from eight to ten millimeters. The flap was attached by means of three silk sutures, while the wound left by the transposition of the flap was united by a continuous catgut suture. The operation was done under strictly antiseptic precautions.

On removing the dressing, four days later, complete union was noticeable, but two days subsequently the epithelium of the flap began to peel off at its distal extremity and the entire transplanted flap changed into an ulcerated surface without extension of the ulceration below the papillary layer. Within some ten days the ulceration had healed, but the scar shrunk again to such an extent that the result of the operation was not nearly as satisfactory as anticipated, although there was quite an improvement over the previous condition.

The attempt to prevent a downward pulling of the lid by applying adhesive plasters traction between the forehead and the cheek for some weeks gave no satisfactory result.

December 4th I repeated the operation, liberating the skin by transverse incision following the edge of the lower lid about two millimeters from the insertion of the cilia, and dissecting the lower lid away from the cicatrix until it resumed its place to a normal extent. In order to fill up the resulting gaping wound, I took a flap this time from the skin of the forearm—of course, without pedicle. As recommended by Wolfe and by E. Meyer, after having antisepticised the arm, a flap considerably larger than the gaping wound was transfixed with a Græfe's cataract knife, and nearly, not but quite completely cut off. A strip of adhesive plaster (gold-beater's skin) was then attached to the flap and the latter cut off entirely and placed on the wound of the eye-lid, where the overlapping adhesive plaster retained it in position without the necessity of sutures.

On the fourth day the dressing was changed and complete union was found. Within the next five days a narrow strip along the inferior border of the transplanted flap became mummified, evidently because it overlapped the healthy skin. When this was detached no ulcerating surface could be seen underneath. The result was therefore a complete success.

[1] Read before the Chicago Medical Society, February 27.

Within the last two months a very slight retraction has again occurred, but not to an extent requiring any further interference.

63 State Street.

